# Integrated bioinformatics analysis and in vivo validation of potential immune-related genes linked to diabetic nephropathy

**DOI:** 10.1016/j.heliyon.2024.e40151

**Published:** 2024-11-05

**Authors:** Jinxiu Deng, Peiwen Wu

**Affiliations:** a Department of Endocrinology, the First Affiliated Hospital of Fujian Medical University, Fuzhou, Fujian, 350005, China; b Department of Nephrology, Longyan First Affiliated Hospital of Fujian Medical University, Longyan, 364000, China; c Department of Endocrinology, National Regional Medical Center, Binhai Campus of the First Affiliated Hospital, Fujian Medical University, Fuzhou, Fujian, 350212, China; d Clinical Research Center for Metabolic Diseases of Fujian Province, the First Affiliated Hospital, Fujian Medical University, Fuzhou, Fujian, 350005, China; e Fujian Key Laboratory of Glycolipid and Bone Mineral Metabolism, the First Affiliated Hospital, Fujian Medical University, Fuzhou, Fujian, 350005, China

**Keywords:** Diabetic nephropathy, Immunodulation-related genes, Bioinformatics, Signaling pathways, Diagnostic efficacy, Immune cell infiltration, Immunotherapy development

## Abstract

**Background:**

Diabetic nephropathy (DN) is a common microvascular complication of diabetes mellitus and the main cause of chronic renal failure. This study explored the potential immunomodulation-related genes (IRGs) in DN using bioinformatics.

**Methods:**

IRGs were identified using GeneCards, and differentially expressed genes were identified using the GSE99339, GSE96804, and GSE30122 datasets. We conducted enrichment analyses using Gene Ontology, gene set enrichment analysis (GSEA), and Kyoto Encyclopedia of Genes and Genomes to identify the associated signaling pathways. Prognostic models were constructed using Least Absolute Shrinkage and Selection Operator regression. The predictive power of IRGs was evaluated using receiver operating characteristic (ROC) curves. Furthermore, we utilized ssGSEA to determine the relative abundance of immune cell infiltration. The expression of five significant IRGs was further validated using immunohistochemistry (IHC) in individuals with DN and real-time PCR (RT-PCR) in animal experiments.

**Results:**

In total, 17 immunomodulation-related differentially expressed genes were identified, which were enriched in immune-associated pathways and inflammation. GSEA unveiled substantial enrichments in metabolic irregularities and the structural composition of the extracellular matrix. ROC analysis results revealed that the diagnostic efficacy of *IFNAR2* and *CASP3* was comparatively high. Notably, we identified potential IRGs for DN, including *CASP3*, *LGALS9*, and *SST*, using IHC and RT-PCR.

**Conclusions:**

*CASP3, LGALS9,* and *SST* are potential IRGs in patients with DN. Our findings may offer a theoretical basis for developing more focused and innovative immunotherapy approaches for patients with DN.

## 1 Introduction

Diabetic nephropathy (DN), which is the primary cause of chronic renal failure, is the most prevalent and dangerous microvascular complication of diabetes mellitus. Approximately 415 million people worldwide had diabetes as of 2015, and projections indicate that this number will surge to 693 million by 2045 [1]. Notably, up to 40 % of individuals with diabetes may develop DN during their lifetime [2]. In most nations, diabetes affects more than 50 % of patients with end-stage renal disease (ESRD) who require renal replacement treatment for DN [3]. Historically, albuminuria or microalbuminuria has served as the primary diagnostic marker for DN. Nevertheless, accumulating data indicate that some patients with DN experience a reduction in renal function, even without showing signs of albuminuria. This clinical presentation suggests that additional factors are required to predict the occurrence and development of DN. Currently, the primary treatment approaches revolve around managing glucose levels and reducing glomerular intracapsular pressure [4]. However, some patients do not benefit from these drugs. Currently, there are no adequate effective response interventions to completely halt the progression of DN to ESRD. Therefore, it is important to identify new diagnostic and therapeutic strategies to delay DN progression. DN is characterized by a complex and multifaceted pathophysiology. Hyperglycemia contributes to glomerular damage by recruiting immune cells, stimulating mesangial proliferation, and triggering the release of inflammatory factors [5]. The study of inflammatory reactions and immune cells involved in the development of DN has been gaining attention [6,7]. Kidney inflammation results from intricate interactions between local renal cells and infiltrating leukocytes [8]. Previous studies have found that activated immune cells are involved in various renal regulatory processes in DN. Moreover, certain immune-related genes are upregulated in the kidney of animals and individuals diagnosed with diabetes [9]. These genes are essential for attracting immune cells. Further understanding of immunomodulatory-related genes (IRGs) could shed new light on the molecular mechanisms of DN and offer fresh insights into potential immunotherapeutic approaches.

In recent years, bioinformatic analysis has been extensively utilized to identify differentially expressed genes (DEGs). In this study, we employed bioinformatics to explore the functions of IRGs in the pathogenesis of DN. The outcomes of this study will contribute to a deeper understanding of how the immune system is involved in the development of DN and provide potential targets for DN therapy and prevention.

## 2 Materials & methods

### 2.1 Data acquisiton

We accessed the Gene Expression Omnibus (GEO) database [10] (https://www.ncbi.nlm.nih.gov/geo/) and retrieved DN microarray data using the R package GEOquery [11]. Specifically, we downloaded the microarray datasets GSE99339 [12], GSE96804 [13], and GSE30122 [14], with GSE99339 and GSE96804 serving as test datasets and GSE30122 as the verification dataset. Detailed information is provided in Table 1.

**Table 1:** Diabetic nephropathy dataset information list.

	GSE99339	GSE96804	GSE30122
Platform	GPL19109, GPL19184	GPL17586	GPL571
Species	*Homo sapiens*	*Homo sapiens*	*Homo sapiens*
Tissue	Glomeruli from kidney biopsy	Glomeruli	Glomeruli and Tubuli
Samples in normal group	0	20	50
Samples in DN group	14	41	19
Reference	[12]	[13]	[14]

DN, diabetic nephropathy.

Using “Immunomodulatory,” “Protein Coding,” and “Relevance score>1” as the selection criteria in the GeneCards database [15], we obtained 193 IRGs. In addition, we searched the Molecular Signatures Database (MSigDB) [16] with the keyword “Immunomodulatory” and obtained 343 IRGs, as detailed in Table 2.

**Table 2:** Immunomodulatory-related genes.

IRGs						
*PIBF1*	*CTLA4*	*ITGA4*	*IRF4*	*F2RL1*	*HNRNPLL*	*PLIN2*
*TNF*	*SEMA7A*	*CRP*	*IL23A*	*ADAM9*	*IER2*	*PLK4*
*IFNG*	*CSF3*	*LTF*	*COMP*	*AREG*	*IFI16*	*PLXDC2*
*IL10*	*CD274*	*DHODH*	*IL11*	*ARHGAP21*	*IFI30*	*PMF1*
*IFNA1*	*MIF*	*CD200*	*MYC*	*ARRDC4*	*IFI44*	*PPP1R16B*
*IL2*	*STAT1*	*TNFRSF11B*	*ENG*	*ATAD5*	*IFIT3*	*PRTN3*
*IL6*	*CD4*0LG	*CXCR3*	*HLA-DQB1*	*BATF*	*IFNGR1*	*PTCH1*
*ITFG1*	*BMP6*	*CASP3*	*VCAM1*	*BCL2L11*	*IL1R2*	*RABGAP1L*
*IL1B*	*TLR9*	*IKZF3*	*HP*	*BRCA1*	*IL1RL1*	*RGS16*
*IFNA2*	*CCL4*	*CUL4A*	*BAX*	*BST1*	*IL6ST*	*RIGI*
*CRBN*	*HGF*	*TNFSF11*	*HLA-A*	*BST2*	*IRF7*	*RNF125*
*CXCL8*	*IL1R1*	*HLA-B*	*IL22*	*CABLES1*	*ISG20*	*ROM1*
*IFNB1*	*SLURP1*	*CXCL10*	*IGHE*	*CAPG*	*ISOC1*	*RPAP3*
*IFNL3*	*HMOX1*	*IL7*	*PRDM1*	*CBLB*	*ITIH5*	*RSAD2*
*IL2RA*	*PDE4A*	*LGALS9*	*HLA-DQA1*	*CCDC134*	*ITM2A*	*RTP4*
*IFNL1*	*VDR*	*MAPK3*	*IL18R1*	*CCNYL1*	*KIF18A*	*SAA3P*
*IL4*	*B2M*	*ISG15*	*PCNA*	*CCR9*	*KLHDC2*	*SERPINB9*
*IFNL2*	*IL12B*	*CD276*	*CYP19A1*	*CCRL2*	*LCLAT1*	*SETBP1*
*IL5*	*S100A7*	*JAK2*	*RORC*	*CD160*	*LITAF*	*SHMT1*
*CSF2*	*IL1F10*	*TNFSF10*	*S100A9*	*CD180*	*MAFF*	*SKAP2*
*IL16*	*NOS2*	*PDCD1*	*SIGLEC5*	*CD38*	*MDFIC*	*SLC25A36*
*IL2RB*	*HLA-DRB1*	*CNR1*	*CSN2*	*CD44*	*METTL2B*	*SOCS4*
*CST9*	*CXCR4*	*CCL3*	*THBS1*	*CDC14A*	*MFSD2A*	*SPATS2*
*TLR4*	*CSF1*	*TLR8*	*IRF1*	*CEP170*	*MIR15A*	*SPC24*
*IDO1*	*JUN*	*ADIPOQ*	*SST*	*CPT1A*	*MPO*	*SPIN2A*
*ICAM1*	*STAT3*	*ADORA2A*	*EIF2S1*	*CYP20A1*	*MS4A3*	*SRGAP3*
*IL1A*	*TLR7*	*XIAP*	*VTCN1*	*DUSP16*	*MT1A*	*STX11*
*IL13*	*PLA2G3*	*BIRC2*	*CLEC12A*	*EEA1*	*MT2A*	*SULT2B1*
*IL18*	*CLEC4E*	*KLK3*	*IL17B*	*EIF2AK2*	*MXD1*	*SYCE2*
*VEGFA*	*TMPO*	*MOG*	*IFNAR2*	*ENTPD1*	*MYB*	*SYT11*
*ITGB2*	*IL3*	*TP53*	*IGF1*	*ETS2*	*NAMPT*	*TAF4B*
*HLA-G*	*TNFRSF1A*	*FAS*	*P2RY11*	*EXO1*	*NDRG1*	*TBC1D19*
*TGFB1*	*FASLG*	*CD69*	*SPHK2*	*F13A1*	*NEB*	*TFDP2*
*LGALS1*	*CD40*	*ADAM17*	*CEACAM6*	*FABP7*	*NEURL3*	*TFEC*
*IL1RN*	*CD80*	*IL6R*	*SERPINB1*	*FAM3C*	*NFIL3*	*TGIF1*
*CAMP*	*LY9*	*MADCAM1*	*HSPA1B*	*FANCC*	*NHLRC3*	*TIGIT*
*CCL5*	*TLR3*	*INS*	*MAPK14*	*FGL2*	*NINJ2*	*TMBIM4*
*LTA*	*PPARG*	*IL1*7RB	*NR1H2*	*FILIP1*	*NR4A1*	*TNFRSF18*
*IL1RAPL2*	*MAPK1*	*LST1*	*CD200R1*	*FPR1*	*OCIAD2*	*TNFRSF4*
*PTGS2*	*MBP*	*CD55*	*TNFRSF1B*	*G6PC3*	*ORC1*	*TNFRSF9*
*PAEP*	*BIRC5*	*BCL2L1*	*CD14*	*GPR146*	*P2RY13*	*TOX*
*CCL2*	*HMGCR*	*C1QA*	*NT5E*	*GSTO1*	*PACSIN1*	*TPD52*
*IKZF1*	*CNR2*	*FOXP3*	*APOE*	*HAUS7*	*PARP12*	*TRAM2*
*IL17A*	*CD4*	*HRH4*	*NR3C1*	*HEG1*	*PBK*	*TRIM5*
*IL15*	*LGALS3*	*GAA*	*CD28*	*HELLS*	*PDE4D*	*TRPC1*
*TLR2*	*CD86*	*CYCS*	*CD79A*	*HEXIM1*	*PDE7B*	*TRPS1*
*EPO*	*DDB1*	*SCGB1A1*	*ITGAM*	*HIF1A*	*PHLDA1*	*TUBGCP2*
*LBR*	*BIRC3*	*DPEP1*	*SIK2*	*HIPK2*	*PI4K2B*	*TWSG1*
*UBASH3B*	*UFD1*	*USP6NL*	*VCAN*	*XCL1*	*ZC3H12C*	

IRG, immunomodulation-related gene.

### 2.2 Differential analysis

DEGs between DN and normal groups were obtained by using the R package sva [17]. We then merged and debatched datasets GSE99339 and GSE96804 into Dataset-DN. We conducted principal component analysis (PCA) [18] on the dataset before and after debatching to assess the impact of debatching. Subsequently, we employed the R package limma [19]for differential analysis, using the grouping information (DN/normal) in Dataset-DN. The DEGs related to immune regulation were obtained by intersecting all DEGs and IRGs based on the criteria of |logFC| >0.5 and P.adj <0.05. We created a volcano plot and heatmaps of DEGs related to immune regulation and of the association between DEGs related to immune regulation. Additionally, we created group comparison charts for Dataset-DN to display the expression trends of DEGs associated with immunoregulation.

### 2.3 Functional enrichment analysis

R package cluster was used to conduct Gene Ontology (GO) and Kyoto Encyclopedia of Genes and Genomes (KEGG) pathway analysis. Gene set enrichment analysis (GSEA) was used to enrich KEGG terms.

### 2.4 Development and building the diagnostic model

We employed Least Absolute Shrinkage and Selection Operator (LASSO) regression to build prognostic diagnostic models and select relevant variables, mitigating overfitting while enhancing model generalization. The glmnet package [20] was used to identify immunomodulation-related differentially expressed genes (IRDEGs) with non-zero coefficients at the optimal lambda value [21]. Subsequently, we used the Support Vector Machine (SVM) [22] algorithm to build the SVM model based on the IRDEGs that achieved the highest accuracy and lowest error rates.

Logistic regression was applied to the IRDEGs selected through LASSO and SVM analyses. A nomogram was established using the R package rms. Multi-factor regression analysis enabled the creation of a calibration curve for precision and sensitivity assessment. We opted for the most suitable model based on receiver operating characteristic (ROC) curves [23].

### 2.5 Functional similarity analysis

To determine the semantic similarity of key genes obtained from Dataset-DN, we employed the GO SemSim package [24] and evaluated the GO semantic similarity. We used the ggplot package to analyze functional similarity among the genes. The Spearman approach was used to investigate the relationships between the genes.

### 2.6 Regulatory networks

We created the miRNA–mRNA–transcription factor (TF) and mRNA–drug interaction networks associated with key genes through four databases: the miRDB, CHIPBase, hTFtarget, and DGIdb database.

### 2.7 GSEA for differential expression in the diagnostic model

First, we applied the limma package to the Dataset-DN dataset, separating it into high- and low-risk groups. A comparative analysis was conducted between the two groups using a volcano plot, followed by GSEA.

### 2.8 Immune infiltration analysis

First, distinct types of infiltrating immune cells were labeled. Then, the enrichment scores representing different immune cell types in each sample were calculated using the ssGSEA [25] algorithm within the GSVA package. Using the ggplot2 package, we integrated the gene expression matrices from Dataset-DN to assess the relationship between immune cells and key genes in the two groups of the logistic regression model (High/Low).

### 2.9 Immunohistochemical staining

Paraffin-embedded tissues were obtained from five individuals clinically and histopathologically diagnosed with DN, while five paracancerous specimens from patients with normoglycemic renal malignancy served as normal controls. These specimens were provided by the Longyan First Affiliated Hospital of Fujian Medical University. The Ethics Committee of the Longyan First Affiliated Hospital of Fujian Medical University provided ethical approval(Approval No:LYREC2023-k085-01), and informed consent for collecting and preserving samples and details was obtained from each patient.The preparation of these samples involved sectioning paraffin-embedded tissues, each 5 μm thick, which were subsequently affixed to glass slides. The slides underwent a baking process at 60 °C for an hour and were then subjected to deparaffinization in Xylene. Following this, a rehydration process was carried out sequentially using 99 %, 95 %, and 70 % ethanol. Subsequently, the primary antibodies, namely NR4A1 (12235-1-AP, Proteintech, 1/200), somatostatin (SST) (17512-1-AP, Proteintech, 1/200), IFNAR2 (DF6605, Affinity, 1/100), and LGALS9 (17938-1-AP, Proteintech, 1/100), were incubated overnight on the tissue sections at 4 °C. Following this incubation, the sections were allowed to incubate with secondary antibodies. To visualize the staining, 3,3′N-diaminobenzidine tetrahydrochloride (CW0125M; CWBIO) was added to the sections and incubated accordingly. Sections were observed and photographed under a microscope (BX43, OLYMPUS). Quantitative analysis was conducted using Image J software.

### 2.10 Animal experiments

Fifteen eight-week-old male C57BL/6JGpt mice (strain no. N000013), weighing approximately 25 g, were provided by GemPharmatech (Nanjing, China). The mice were randomly assigned to either the DN group (n = 15) or the normal group (n = 5). The DN group received 50 mg/kg of streptozotocin (STZ) to induce hyperglycemia [26]. Mice were considered diabetic if their postprandial blood glucose level was 200–300 mg/dl one week after the STZ injection. The DN group was defined by a urine protein level exceeding 30 mg/1d. After 12 weeks, kidney samples were collected after mice were euthanized. The animal studies were approved by the Ethics Committee of Fujian Medical University (Approval No: IACUC FJMU 2023-Y-1059).

### 2.11 RNA extraction and real-time PCR (RT-PCR) testing

RNA extraction was performed using the RNA Simple Total RNA Kit (TIANGEN, DP419), and the isolated RNA was reverse transcribed into cDNA using the Hifair® III 1st Strand cDNA Synthesis SuperMix for RT-PCR (Yeasen Biotechnology, 11141ES60). RT-PCR was conducted using a Roche LightCycler® 96 Instrument, and the RT-PCR threshold cycle values were measured using LightCycler® software. In this study, comparative gene expression was estimated as 2^−ΔΔCt^. The primers used were as follows: *CASP3* (F: 5′-CTCGCTCTGGTACGGATGTG-3′, R: 5′-TCCCARAAATGACCCCTTCATCA-3′); *IFNAR2* (F: 5′-TGTCTGCGAGCCTAGAGACTA-3′, R: 5′-AGCCGGGAATTTCGTATTGTTAT-3′); *LGALS9* (F: 5′-TTACTGGACCAATCCAAGGAGG-3′, R: 5′-AGCTGTTCTGAAAGTTCACCAC-3′); *NR4A1* (F: 5′-GAGTTCGGCAAGCCTACCAT-3′, R: 5′-GTGTACCCGTCCATGAAGGTG-3′); *SST* (F: 5′-CCACCGGGAAACAGGAACTG-3′, R: 5′-TTGCTGGGTTCGAGTTGGC-3′)

### 2.12 Statistical analysis

R (version 4.2.3) was employed for all statistical analyses. Continuous variables are presented as mean ± standard deviation. The Wilcoxon rank-sum test was used to compare continuous variables between two groups, while more than two groups were compared using the Kruskal–Wallis test. Fisher's exact test or the chi-square test was used for statistical significance analysis of categorical data. When no further parameters were supplied, the results were computed using Spearman's correlation analysis between the distinct molecules and all P-statistics.

## 3 Results

### 3.1 Analysis flow chart

**Fig. 1:**
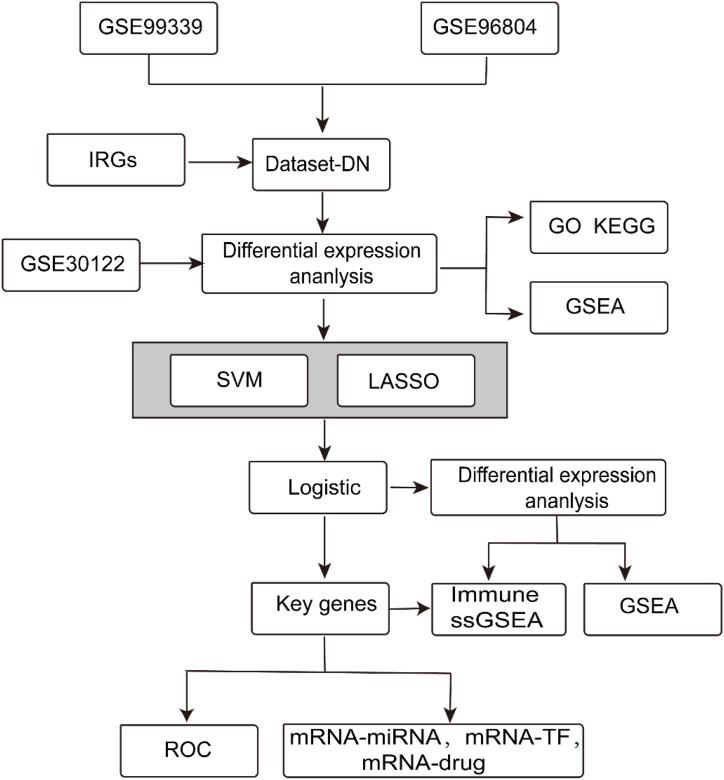
Technology roadmap. DN, diabetic nephropathy; GO, Gene Ontology; GSEA, gene set enrichment analysis; IRG, immunomodulation-related gene; KEGG, Kyoto Encyclopedia of Genes and Genomes; LASSO, Least Absolute Shrinkage and Selection Operator; ROC, receiver operating characteristic; SVM, support vector machine; TF, transcription factor.

The technical pathway for this bioinformatics analysis is illustrated in Fig. 1.

### 3.2 Difference analysis of dataset-DN

**Fig. 2:**
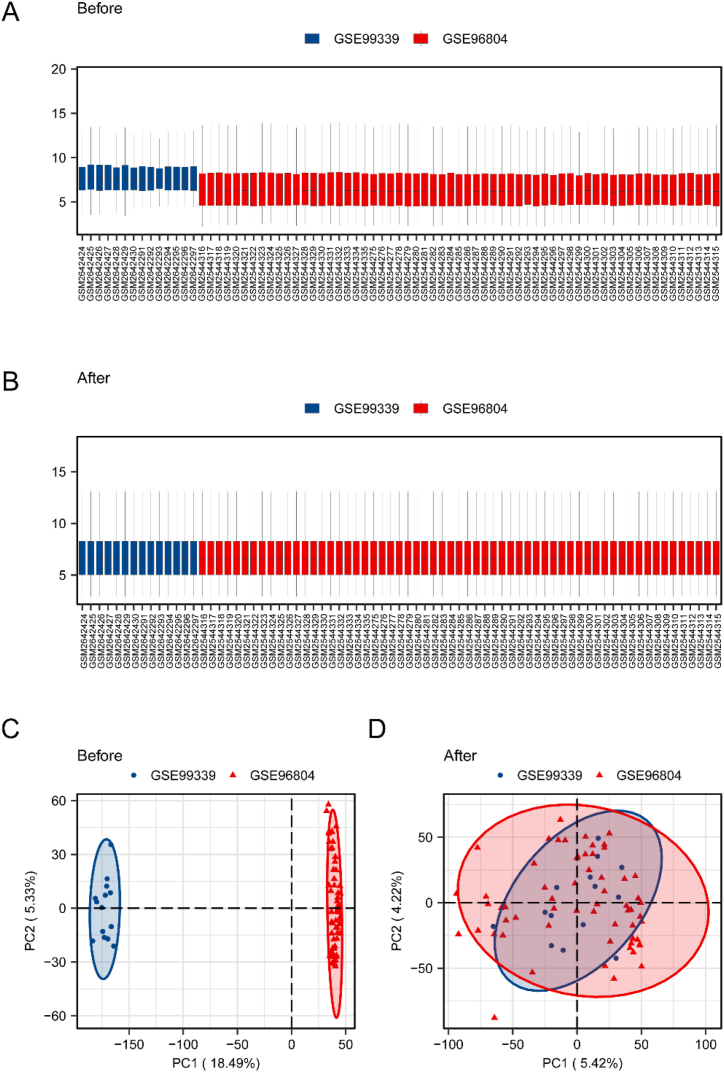
Data set merge correction. A. Dataset-DN box plot before correction. B. Box plot of corrected Dataset-DN. C. Dataset–dataset-DN PCA map before adjustment. D. Dataset: DN PCA map of the corrected dataset. DN, diabetic nephropathy; PCA, principal component analysis.

We initially employed the R package sva to create the combined GEO dataset–Dataset-DN. The effectiveness of batch removal was confirmed through distribution boxplots and PCA graphs (Fig. 2A–D).

**Fig. 3:**
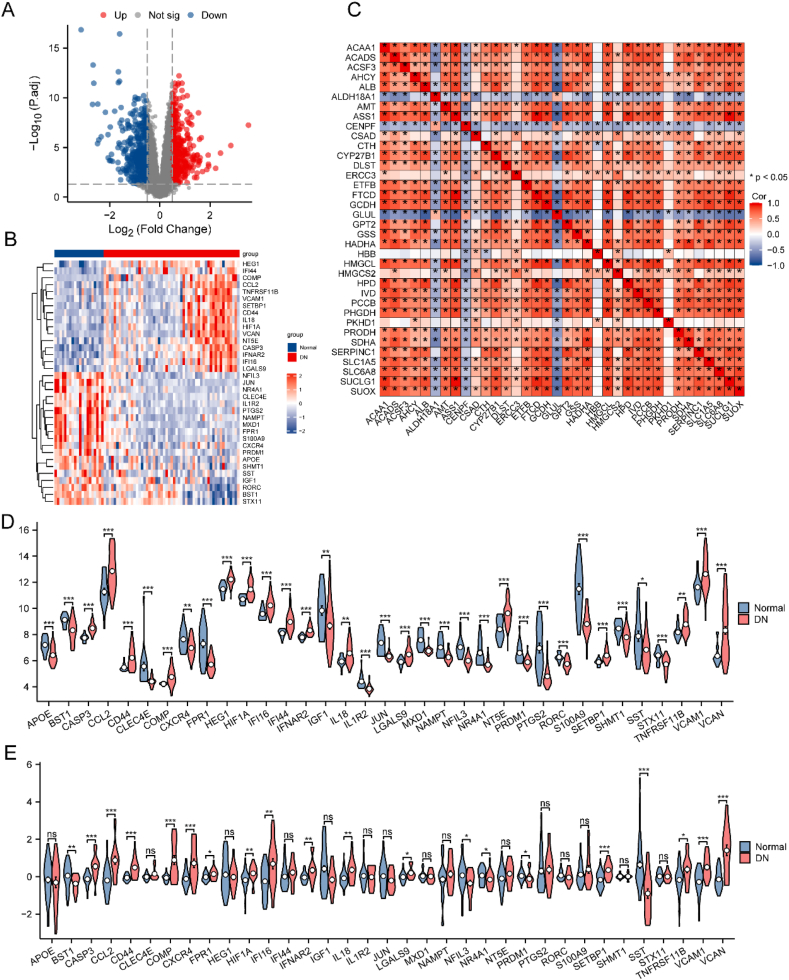
Difference analysis of Dataset-DN. A. Volcano plot of DEGs in Dataset-DN. B. Heat map of 35 DEGs related to immune regulation in Dataset-DN. C. Correlation heatmap of 35 DEGs related to immune regulation. D. Group comparison chart of IRDEGs in Dataset-DN. E. Group comparison chart of IRDEGs in dataset GSE30122. ∗ denotes statistical significance at P < 0.05, ∗∗ denotes statistical significance at P < 0.01, and ∗∗∗ denotes statistical significance at P < 0.001. DN, diabetic nephropathy; DEG, differentially expressed genes.

A total of 761 DEGs meeting the criteria of |logFC| >0.5 and P.adj <0.05 were identified in Dataset-DN during the differential analysis. Visualization of these results included the creation of volcano plots using the R package ggplot2 (Fig. 3A). Further, we identified an intersection of DEGs and IRGs, resulting in a total of 35 DEGs related to immune regulation. These genes included *APOE, BST1, CASP3, CCL2, CD44, CLEC4E, COMP, CXCR4, FPR1, HEG1, HIF1A, IFI16, IFI44, IFNAR2, IGF1, IL18, IL1R2, JUN, LGALS9, MXD1, NAMPT, NFIL3, NR4A1, NT5E, PRDM1, PTGS2, RORC, S100A9, SETBP1, SHMT1, SST, STX11, TNFRSF11B, VCAM1*, and *VCAN*. A heatmap was constructed using R (Fig. 3B), and a correlation heat map was employed to visualize the interaction between the IRDEGs (Fig. 3C). The majority of IRDEGs exhibited positive associations.

Additionally, we created a group comparison chart to display the expression of 35 IRDEGs in Dataset-DN (Fig. 3D) and GSE30122 (Fig. 3E). In the GSE30122 dataset, most of the IRDEGs also showed statistically significant expression differences. Subsequently, 17 genes, including BST1, *CASP3, CCL2, COMP, HIF1A, IFI16, IFNAR2, IL18, LGALS9, NFIL3, NR4A1, PRDM1, SETBP1, SST, TNFRSF11B, VCAM1,* and *VCAN*, were selected.

### 3.3 GO/KEGG

**Fig. 4:**
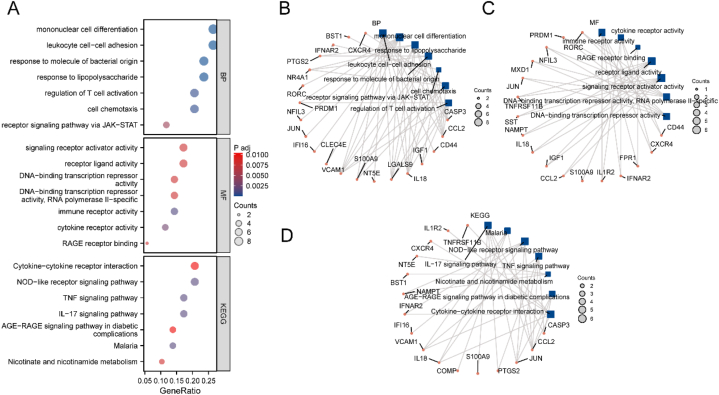
GO/KEGG enrichment analysis. A. Outcomes of the GO enrichment analysis. B–D. BP divergence network diagram of the GO/KEGG enrichment analysis. (B). The MF divergence network diagram of GO (C). Divergence network graph of KEGG (D). The screening criteria included a significantly adjusted P-value (P.adj) of less than 0.05 and a false discovery rate (FDR) value (q-value) of less than 0.05. GO, Gene Ontology; KEGG, Kyoto Encyclopedia of Genes and Genomes.

We performed GO/KEGG functional enrichment analysis on the 17 IRDEGs (Table 3). The screening standards were set at P.adj <0.05, with an FDR value (q-value) of <0.05. The results were visualized using a bubble plot (Fig. 4A). The GO/KEGG analysis revealed that IRDEGs exhibited enrichment in various biological processes, including processes related to mononuclear cell differentiation, leukocyte adhesion to other cells, signaling via the JAK–STAT receptor pathway, and other BP pathways (Fig. 4B). Additionally, these IRDEGs were associated with processes such as the activation of signaling receptors, ligand activity, repression of DNA transcriptional activity, and specific inhibition of RNA polymerase II activity. They were also linked to cytokine receptor activity, RAGE receptor binding, and other MF correlations (Fig. 4C).

**Table 3:** GO/KEGG enrichment analysis results of IRDEGs.

Ontology	ID	Description	Gene Ratio	Bg Ratio	P.adj	q-value
BP	GO:1903131	Mononuclear cell differentiation	9/34	433/18800	5.15E-05	2.83E-05
BP	GO:0007159	Leukocyte cell–cell adhesion	9/34	381/18800	3.43E-05	1.89E-05
BP	GO:0002237	Response to molecule of bacterial origin	8/34	354/18800	8.42E-05	4.64E-05
BP	GO:0032496	Response to lipopolysaccharide	8/34	333/18800	7.03E-05	3.87E-05
BP	GO:0050863	Regulation of T cell activation	7/34	342/18800	0.00048	0.000263
BP	GO:0060326	Cell chemotaxis	7/34	315/18800	0.00041	0.000226
BP	GO:0007259	Receptor signaling pathway via JAK–STAT	4/34	173/18800	0.00494	0.002723
MF	GO:0048018	Receptor ligand activity	6/35	489/18410	0.00804	0.004833
MF	GO:0030546	Signaling Receptor activator activity	6/35	496/18410	0.00804	0.004833
MF	GO:0140375	Immune receptor activity	5/35	148/18410	0.00135	0.000812
MF	GO:0001227	DNA-binding transcription repressor activity, RNA polymerase II-specific	5/35	321/18410	0.00804	0.004833
MF	GO:0001217	DNA-binding transcription repressor activity	5/35	325/18410	0.00804	0.004833
MF	GO:0004896	cytokine receptor activity	4/35	97/18410	0.00269	0.00162
MF	GO:0050786	RAGE receptor binding	2/35	10/18410	0.00804	0.004833
KEGG	hsa04621	NOD-like receptor signaling pathway	6/29	184/8164	0.00157	0.001142
KEGG	hsa04060	Cytokine–cytokine receptor interaction	6/29	295/8164	0.01041	0.007562
KEGG	hsa04657	IL-17 signaling pathway	5/29	94/8164	0.00157	0.001142
KEGG	hsa04668	TNF signaling pathway	5/29	112/8164	0.00157	0.001142
KEGG	hsa05144	Malaria	4/29	50/8164	0.00157	0.001142
KEGG	hsa04933	AGE–RAGE signaling pathway in diabetic complications	4/29	100/8164	0.01028	0.007469
KEGG	hsa00760	Nicotinate and nicotinamide metabolism	3/29	36/8164	0.00824	0.005991

GO, Gene Ontology; IRDEG, immunomodulation-related differentially expressed gene; KEGG, Kyoto Encyclopedia of Genes and Genomes.

The enriched KEGG pathways primarily encompassed cytokine–cytokine receptor interactions, nucleotide-binding oligomerization domain (NOD)-like receptor signaling, the TNF signaling cascade, the IL-17 signaling pathway, the advanced glycation end (AGE)–receptor for AGE (RAGE) signaling pathways implicated in diabetic complications, malaria, nicotinate metabolism, and nicotinamide metabolism (Fig. 4D).

### 3.4 GSEA

**Fig. 5:**
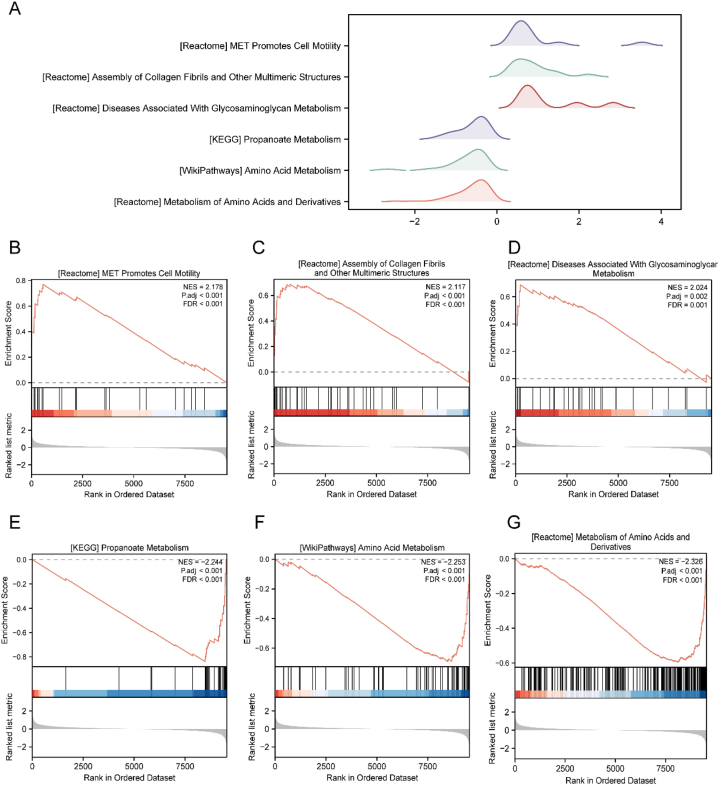
GSEA of Dataset-DN. A. DN/normal grouping sample groups of Dataset-DN. B–G. The genes between the DN/normal grouping sample groups in Dataset-DN were significantly enriched in MET activation, promoting cell motility (B), assembly of collagen fibrils and other multimeric structures (C), diseases associated with glycosaminoglycan metabolism (D), propanoate metabolism (E), amino acid metabolism (F), metabolism of amino acids and derivatives (G), and other pathways. DN, diabetic nephropathy; GSEA, gene set enrichment analysis.

We utilized GSEA to investigate gene expression and associated organisms in the DN/normal grouping samples of Dataset-DN. P < 0.05 and an FDR value (q-value) <0.25 were set as the filtering criteria for significant enrichment. GSEA identified significant gene enrichment in various pathways, including MET activation promoting cell motility, collagen fibril assembly, glycosaminoglycan metabolism-related diseases, propanoate metabolism, and amino acid metabolism (Fig. 5A–G, Table 4) in the DN/normal groupings of the Dataset-DN dataset.

**Table 4:** GSEA of Dataset-DN (DN/normal).

ID	Set Size	Enrichment Score	NES	P-value	P.adj	q-value
Reactome metabolism of amino acids and derivatives	217	−0.59514	−2.32585	1.00E-10	3.21E-08	2.60E-08
WP amino acid metabolism	64	−0.6909	−2.25274	2.12E-08	2.97E-06	2.41E-06
KEGG propanoate metabolism	25	−0.83917	−2.24359	6.58E-08	7.38E-06	6.00E-06
Reactome fatty acid metabolism	115	−0.61852	−2.20845	2.46E-09	4.25E-07	3.46E-07
KEGG arginine and proline metabolism	38	−0.74073	−2.16052	4.90E-07	3.93E-05	3.19E-05
WP fatty acid beta oxidation	29	−0.78605	−2.15412	1.17E-06	7.32E-05	5.94E-05
Reactome peroxisomal lipid metabolism	20	−0.83134	−2.1194	2.96E-06	0.000166	0.000135
KEGG tryptophan metabolism	28	−0.77487	−2.11387	4.51E-06	0.000235	0.000191
KEGG fatty acid metabolism	32	−0.75489	−2.11137	6.79E-06	0.000317	0.000258
KEGG butanoate metabolism	29	−0.75357	−2.06513	1.48E-05	0.000614	0.000499
Reactome mitochondrial fatty acid beta oxidation	25	−0.77072	−2.0606	1.70E-05	0.000669	0.000544
WP PI3Kakt signaline pathway	248	0.407307	1.668454	3.27E-05	0.001094	0.000889
WP focal adhesion PI3KAKTMTOR signaling pathway	235	0.408886	1.670875	2.09E-05	0.000721	0.000586
Reactome glycosaminoglycan metabolism	90	0.471831	1.686786	0.001434	0.02174	0.017663
KEGG TGF beta signaling pathway	69	0.491355	1.687937	0.002647	0.033003	0.026813
WP overview of proinflammatory and profibrotic mediators	58	0.530191	1.738385	0.002293	0.030088	0.024446
Reactome pkmts methylate histone lysines	26	0.625333	1.750927	0.003625	0.039629	0.032197
WP TGF beta signaling in thyroid cells for epithelial mesenchymal transition	17	0.729332	1.834464	0.001955	0.026645	0.021648
Reactome keratan sulfate keratin metabolism	27	0.657077	1.857579	0.001071	0.017934	0.014571
Reactome anchoring fibril formation	12	0.821755	1.906227	0.000264	0.005745	0.004667
WP inflammatory response pathway	20	0.733111	1.908861	0.000506	0.009879	0.008026
Reactome diseases associated with glycosaminoglycan metabolism	32	0.687843	2.024478	6.02E-05	0.001776	0.001443
Reactome assembly of collagen fibrils and other multimeric structures	40	0.687766	2.11727	6.51E-06	0.000311	0.000253
Reactome MET promotes cell motility	27	0.770376	2.177878	4.17E-06	0.000223	0.000181

DN, diabetic nephropathy; GSEA, gene set enrichment analysis; KEGG, Kyoto Encyclopedia of Genes and Genomes.

### 3.5 Development of a diagnostic model

**Fig. 6:**
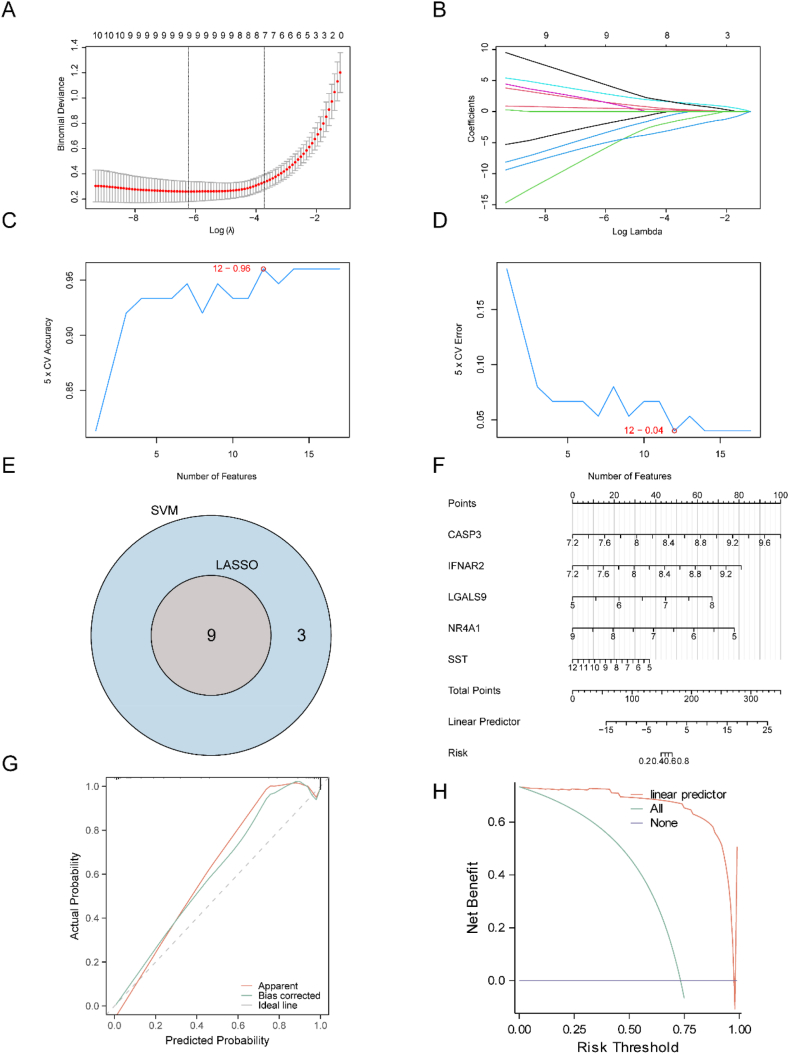
Construction and SVM analysis of the LASSO model of IRDEGs. A. Key gene LASSO regression prognostic model diagram. B. The LASSO regression diagnostic model's variable trajectory diagram. C. The quantity of genes having the highest SVM algorithm accuracy rate. D. Genes with the lowest error rate as identified by the SVM algorithm. E. Venn diagram, intersection of LASSO regression, and SVM algorithm screening genes. F. Nomogram displaying the logistic regression model for predictive value. G. The calibration curve for the logistic predictive value scoring model, indicating good diagnostic performance. H. DCA chart. IRDEG, immunomodulation-related differentially expressed gene; LASSO, Least Absolute Shrinkage and Selection Operator; SVM, support vector machine.

We created a diagnostic model of IRDEGs via LASSO regression analysis (Fig. 6A). Furthermore, we generated a LASSO variable trajectory map and presented the LASSO regression results (Fig. 6B). The LASSO diagnostic model consisted of nine IRDEGs: *HIF1A, NR4A1, IFNAR2, LGALS9, NFIL3, TNFRSF11B, PRDM1, SST*, and *CASP3.* Simultaneously, we built an SVM model based on the 17 IRDEGs and the SVM algorithm to determine the number of genes with the highest accuracy (Fig. 6C) and the lowest error rate (Fig. 6D). The findings demonstrated that the SVM model achieved maximum accuracy when considering 12 genes (*CASP3, PRDM1, NFIL3*, *IFNAR2, TNFRSF11B, NR4A1, LGALS9, HIF1A*, *SST*, *BST1*, *IFI16*, and *IL18*). We intersected the genes obtained using the two algorithms and identified nine intersecting IRDEGs (*HIF1A, NR4A1*, *IFNAR2*, *LGALS9*, *NFIL3*, *TNFRSF11B*, *PRDM1*, *SST*, and *CASP3)*.

We performed logistic regression analysis on the nine IRDEGs obtained from the intersection of the LASSO and SVM algorithms and constructed a logistic regression model. After removing factors with collinearity or variables that led to binary outcomes, we narrowed it down to five key genes: *CASP3, IFNAR2, LGALS9, NR4A1*, and *SST*. Next, we created a nomogram of the logistic predictive value and performed nomogram analysis to assess the model's diagnostic capacity (Fig. 6F). In addition, we conducted a calibration analysis to evaluate the prognostic nomogram generated from the logistic regression model and plotted a calibration curve using the predicted values (Fig. 6G), drawing the optimal theoretical probability (solid line) under different conditions according to the figure. The fit-to-model-predicted probabilities (dashed line) assessed how well the model predicts actual outcomes. Finally, we employed DCA to assess the logistic regression model's logistic predictive value in diagnosing the condition and presented the findings (Fig. 6H). The constructed model exhibited high accuracy in diagnosing the incidence of DN, as shown in Fig. 6G and H.

### 3.6 Diagnostic ROC

**Fig. 7:**
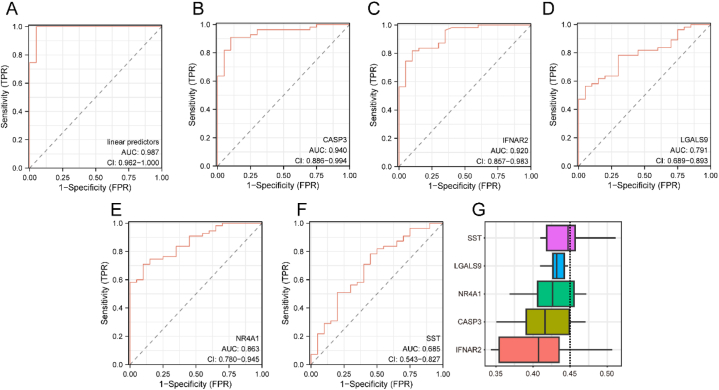
ROC curve and functional similarity analysis. A. The ROC curve results of the logistic regression model linear predictors. B–F. ROC curve results for the five key genes (*CASP3, IFNAR2, LGALS9, NR4A1*, and *SST)*, with Normal and DN as outcome variables. G. Functional similarity analysis. DN, diabetic nephropathy; ROC, receiver operating characteristic.

We combined the logistic regression model linear predictors, involving the five key genes (*CASP3, IFNAR2, LGALS9, NR4A1*, and *SS*T), with the Dataset-DN dataset and plotted the ROC curve (Fig. 7A–F). The ROC curve outcomes indicated that the diagnostic accuracy of the logistic linear predictors was excellent (AUC = 0.987). Specifically, CASP3 and IFNAR2 demonstrated high diagnostic accuracy (AUC >0.9), while LGALS9 and NR4A1 exhibited a moderate degree of accuracy (AUC: 0.7–0.9), and SST's diagnostic accuracy was relatively lower (AUC: 0.5–0.7)

To explore the functional similarity among these five key genes (*CASP3, IFNAR2, LGALS9, NR4A1,* and *SST*), we conducted a functional similarity analysis and visualized the outcomes using box plots (Fig. 7G). Notably, *SST* showed the highest functional similarity score among these key genes.

### 3.7 Regulatory networks

**Fig. 8:**
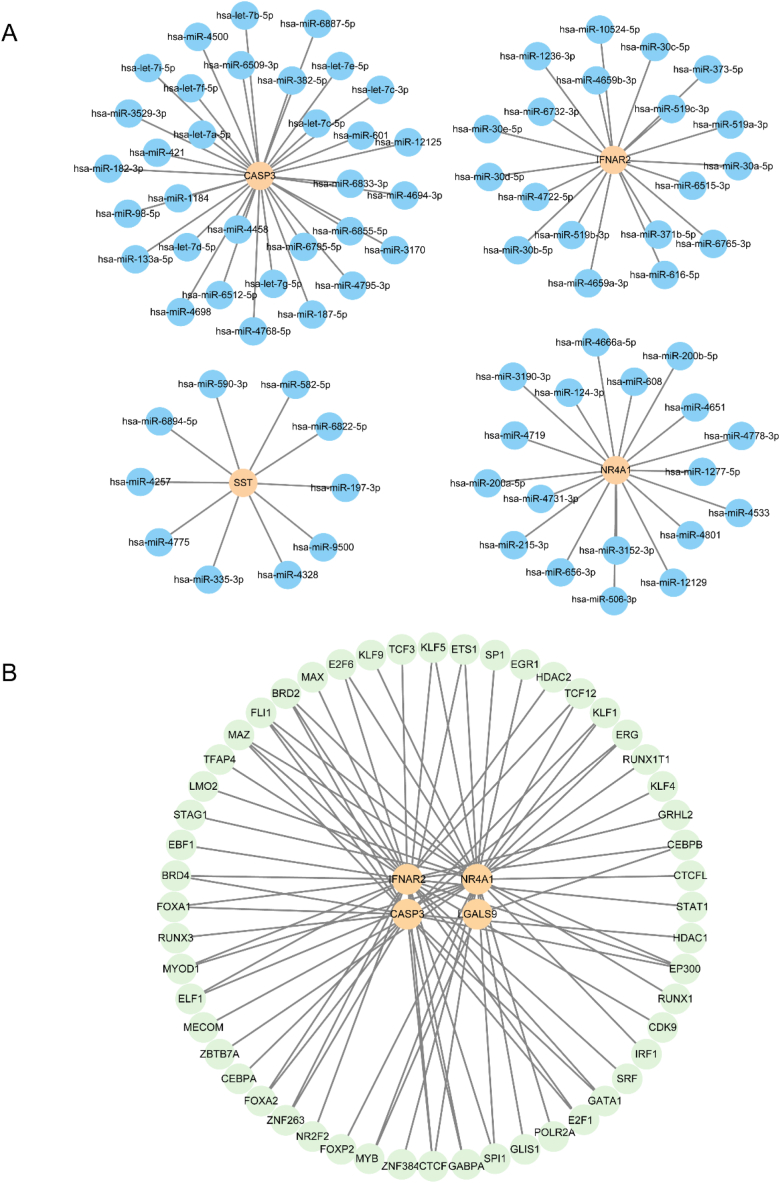
mRNA–miRNA and mRNA–TF interaction networks A. The mRNA–miRNA interaction network of key genes. B. The mRNA–TF interaction network of key genes. (A) The yellow circles in the interaction network represent mRNAs, whereas the blue circles represent miRNAs. (B) The yellow circle in the interaction network represents mRNA, while the green circle represents TF. TF, transcription factor.

For insights into regulatory networks, we utilized the miRDB database's mRNA–miRNA data to predict miRNAs interacting with the five key genes (*CASP3, IFNAR2, LGALS9, NR4A1*, and *SST*). We selected data components with a target score >80 to design an mRNA–miRNA interaction network using Cytoscape software (Fig. 8A). This interaction network comprised four key genes (*CASP3, IFNAR2, NR4A1,* and *SST*), totaling 79 mRNA–miRNA interaction connections. The unique mRNA–miRNA interactions are shown in Table S1.

We identified TFs binding to five key genes by searching the CHIPBase and hTFtarget databases. By intersecting the interactions found in both databases, we isolated four key genes (*CASP3, IFNAR2, LGALS9*, and *NR4A1*) and 52 TFs, resulting in a total of 80 pairs of mRNA–TF interaction relationships, visualized using Cytoscape software (Fig. 8B). The precise mRNA–TF interaction relationships are presented in Table S2.

**Fig. 9:**
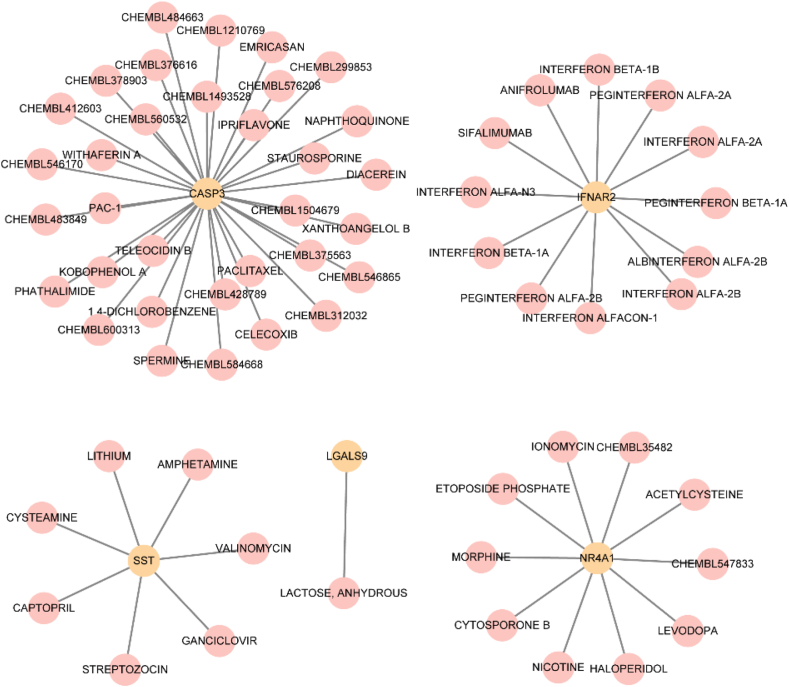
mRNA–drug interaction network The mRNA–drug interaction network, where the yellow circles represent mRNAs and the pink circles represent drugs or molecular compounds.

Finally, we used the DGIdb database to identify potential medicines or chemical compounds associated with the key genes. We found 63 connections to prospective drugs or chemical compounds linked to the five key genes (*CASP3, IFNAR2, LGALS9, NR4A1*, and SST), represented in the mRNA–medication interaction network (Fig. 9). Precise mRNA–medication interaction relationships are presented in Table S3.

### 3.8 Differential expression and GSEA of high-risk and low-risk groups in the diagnostic model

First, we categorized the DN samples into high- and low-risk groups (high/low) according to the predicted values of the logistic regression model. The scoring algorithm for computing the predicted values (linear predictors) was as follows:

linearpredictors=(5.66833960∗CASP3Expression)+(5.431756410∗IFNAR2Expression)+(3.29287062∗LGALS9Expression)+(−2.86340634∗NR4A1Expression)+(−0.77697749∗SSTExpression)

**Fig. 10:**
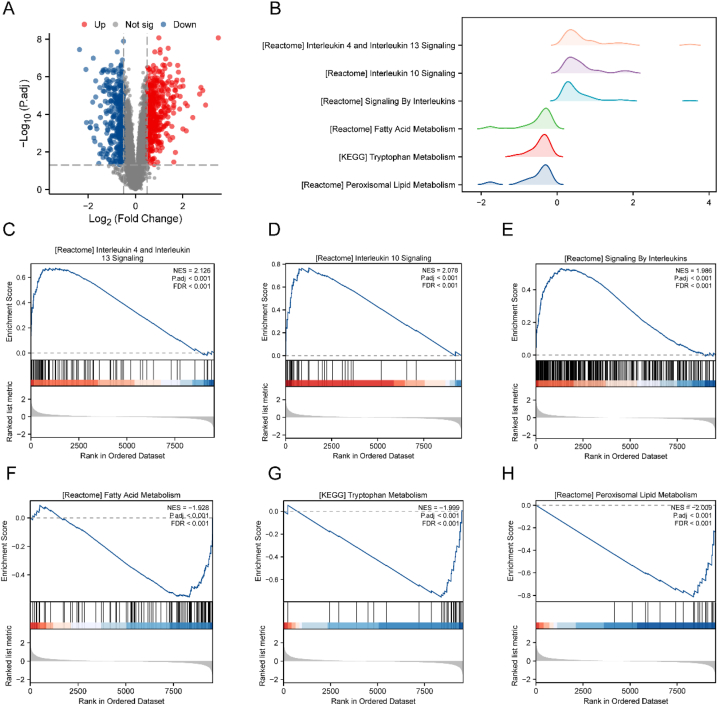
Difference analysis and GSEA of high-risk and low-risk groups. A. DEG analysis volcano plot of Dataset-DN comparing two groups (high/low). B. Six biological features make up the majority of the GSEA between the high/low-grouping sample groups of Dataset-DN. C–H. Interleukin 4 and interleukin 13 signaling, interleukin 10 signaling (D), signaling by interleukins (E), fatty acid metabolism (F), tryptophan metabolism (G), peroxisomal lipid metabolism (H). GSEA, gene set enrichment analysis.

We utilized the limma package to perform a differential analysis on the DN samples in Dataset-DN. This analysis identified a total of 713 DEGs with |logFC| >0.5 and P.adj <0.05. Under this threshold, 387 DEGs were highly expressed in the high group, while 326 DEGs were expressed at low levels in the high group. Differential analysis results are displayed as volcano plots (Fig. 10A).

We then explored the associations between gene expression in the two grouping samples of the Dataset-DN and their corresponding BP, CC, and MF. Significant enrichment was considered at threshold values of P.adj <0.05 and FDR value (q-value) <0.25. The results showed that genes between the high- and low-grouping sample groups of Dataset-DN were significantly enriched in interleukin 4 and interleukin 13 signaling (Fig. 10C), interleukin 10 signaling (Fig. 10D), signaling by interleukins (Fig. 10E), fatty acid metabolism (Fig. 10F), tryptophan metabolism (Fig. 10G), and peroxisomal lipid metabolism (Fig. 10B–H, Table 5).

**Table 5:** GSEA of Dataset-DN (high/low).

ID	Set Size	Enrichment Score	NES	P-value	P.adj	q-value
Reactome peroxisomal lipid metabolism	20	−0.81329	−2.00882	1.86E-05	0.000758	0.000633
KEGG tryptophan metabolism	28	−0.75308	−1.9992	2.27E-05	0.000862	0.000719
Reactome fatty acid metabolism	115	−0.55582	−1.92761	2.05E-06	0.000154	0.000128
KEGG butanoate metabolism	29	−0.70011	−1.86871	0.000247	0.005438	0.004536
KEGG Glycine serine and threonine metabolism	27	−0.70421	−1.86061	0.000263	0.00557	0.004645
KEGG fatty acid metabolism	32	−0.6767	−1.84928	0.000847	0.013576	0.011323
KEGG arginine and proline metabolism	38	−0.65171	−1.84926	0.000385	0.007314	0.006101
KEGG beta alanine metabolism	18	−0.75385	−1.82057	0.000959	0.014942	0.012462
Reactome metabolism of amine derived hormones	13	−0.81304	−1.79652	0.000549	0.009619	0.008022
Reactome sulfur amino acid metabolism	20	−0.72687	−1.79535	0.001589	0.022561	0.018817
WP mitochondrial long chain fatty acid beta oxidation	16	−0.76315	−1.76892	0.001478	0.021395	0.017844
WP amino acid metabolism	64	−0.55589	−1.76859	0.000828	0.013367	0.011149
WP focal adhesion PI3KATAMOTOR signaling pathway	235	0.484558	1.760397	7.96E-06	0.000406	0.000339
WP IL1 and megakaryocytes in obesity	19	0.750121	1.775696	0.00342	0.04018	0.033511
PID IL12 STAT4 pathway	29	0.69176	1.81148	0.001283	0.019191	0.016006
Reactome selenoamino acid metabolism	39	0.667639	1.853923	0.000757	0.012406	0.010347
WP TGFβ signaling in thyroid cells for epithelial mesenchymal transition	17	0.806999	1.87486	0.000301	0.006254	0.005216
PID IL4 pathway	49	0.657128	1.881237	0.00025	0.005438	0.004536
WP overview of proinflammatory and profibrotic mediators	58	0.636864	1.890175	0.000111	0.002932	0.002445
Reactome diseases associated with glycosaminoglycan metabolism	32	0.722498	1.923477	0.00036	0.007023	0.005857
WP inflammatory response pathway	20	0.803976	1.93002	0.00014	0.003469	0.002893
WP IL18 signaling pathway	200	0.554474	1.965542	2.57E-08	4.11E-06	3.43E-06
Reactome signaling by interleukins	334	0.531155	1.985745	1.00E-10	5.61E-08	4.68E-08
Reactome interleukin 10 signaling	35	0.766925	2.078416	3.81E-06	0.000238	0.000198
Reactome interleukin 4 and interleukin 13 signaling	82	0.673777	2.126439	7.88E-08	1.18E-05	9.83E-06

DN, diabetic nephropathy; GSEA, gene set enrichment analysis; KEGG, Kyoto Encyclopedia of Genes and Genomes.

### 3.9 Immune infiltration analysis

**Fig. 11:**
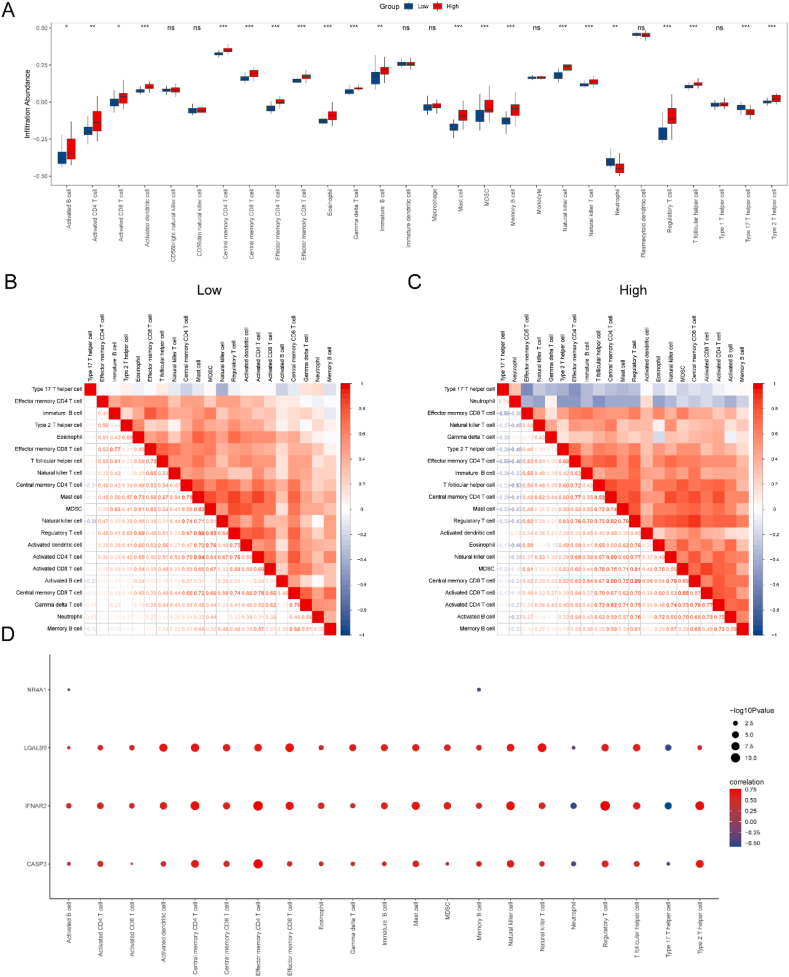
Immune cell infiltration analysis of Dataset-DN samples. A. Comparative visualization of the ssGSEA immune infiltration analysis results between the high- and low-risk groups in the Dataset-DN sample diagnostic model. B–C. Results of the correlation analysis between immune cell infiltration abundance and the two groups in Dataset-DN: (B) low-risk group and (C) high-risk group. D. Bubble diagram illustrating the correlation between immune cells and key genes in Dataset-DN. ∗ denotes statistical significance at P < 0.05, ∗∗ denotes statistical significance at P < 0.01, and ∗∗∗ denotes statistical significance at P < 0.001. DN, diabetic nephropathy.

**Fig. 12:**
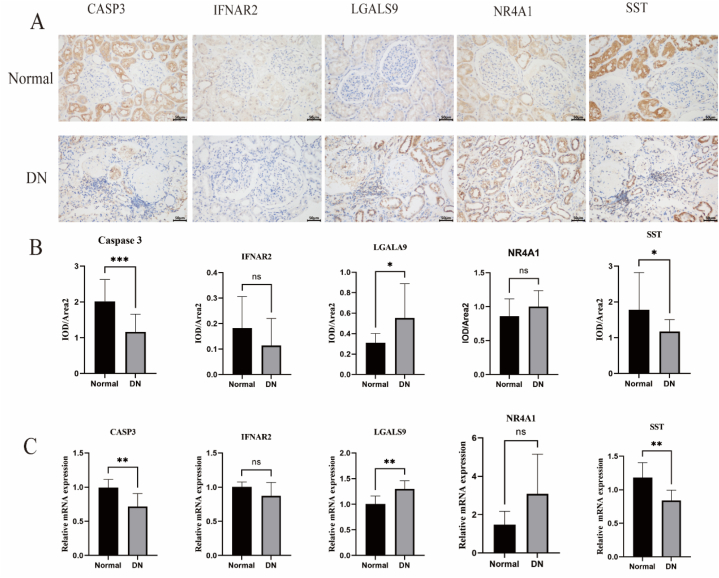
Verification of key genes in patients with DN and animal experiments. (A) IHC staining was utilized to examine the expression of CASP3, IFNAR2, LGALS9, NR4A1, and SST in five paracarinoma kidney tissues (Normal) and 5 DN kidney tissues (scale bar, 50um; magnification, 400 × ). (B) IHC quantitative expression statistics (mean s.d., n = 5). (C) The relative mRNA expression of *CASP3, IFNAR2, LGALS9, NR4A1*, and *SST* in mouse kidney tissue. ∗ denotes statistical significance at P < 0.05, ∗∗ denotes statistical significance at P < 0.01, and ∗∗∗ denotes statistical significance at P < 0.001. DN, diabetic nephropathy; IHC, immunohistochemistry.

In Dataset-DN, the ssGSEA approach was employed to assess the differences in 28 types of immune cells between the Normal and DN groups within the high/low groups. The degree of variation in the infiltration of 28 types of immune cells between distinct high/low groups was analyzed using the Mann–Whitney *U* test, and the results were visualized in group comparison charts (Fig. 11A). Notably, the infiltration abundance of 21 types of immune cells showed significant differences between the two groups. We then evaluated the correlation between the abundance of these 21 immune cell types and the observed statistical differences between the groups (Fig. 11B and C). Additionally, the low-risk group in the Dataset-DN exhibited the presence of regulatory immune cells. In the Dataset-DN high-risk group, the most robust positive relationship was observed between T cells and mast cells (Fig. 11B), while the positive association between regulatory T cells and central memory CD8^+^ T cells was most pronounced (Fig. 11C) (see Fig. 12).

Finally, we examined the correlation between the infiltration abundance of these 21 immune cells and the expression levels of the five key genes (*CASP3, IFNAR2, LGALS9, NR4A1*, and *SST*) screened with P < 0.05, and presented the results in a bubble diagram (Fig. 11D). This analysis highlighted a significant correlation between the abundance of immune cells and the expression of key genes. Specifically, *CASP3, IFNAR2*, and *LGALS9* exhibited positive correlations with most immune cell types.

### 3.10 Verification of key genes

Immunohistochemical (IHC) results from kidney biopsies demonstrated significant statistical differences in the expression of CASP3, LGALS9, and SST between the DN group and the normal group. These results were consistent with the relative mRNA expression in mice.

## 4 Discussion

DN, a common systemic microvascular complication linked to diabetes, has emerged as a leading cause of chronic renal failure on a global scale due to the increasing prevalence of diabetes [1,27]. Managing DN, once it progresses to chronic renal failure, presents even greater challenges compared to other kidney diseases due to its intricate metabolic disorders [28]. Recent research has shed light on the pivotal role of inflammation in both the onset and progression of DN [5,28,29]. Therefore, it is critical to screen immune-related biomarkers for early diagnosis and develop novel therapeutic strategies to postpone DN progression and prevent kidney function deterioration.

In this study, we employed bioinformatic analysis to identify IRDEGs and pathways in patients with DN. We analyzed datasets, GSE99339, GSE96804, and GSE30122. Subsequent GO and KEGG enrichment analyses revealed that the identified IRDEGs were significantly enriched in various biological processes and pathways. These findings are consistent with those of previous studies, suggesting that different types of activated leukocytes and B and T lymphocytes are crucial for the development of DN [30]. The stimulation of different types of immune cells demonstrates the crucial of immunological response as well as regulation in the progression of DN. Cytokines and chemokines are important inflammatory mediators involved in the progression of interstitial inflammation in DN [9]. Moreover, TNF signaling pathway is essential to the occurrence of DN [9,31]. Certain immune cell types, including T cells, neutrophils, macrophages, and dendritic cells, have the capacity to produce the cytokine IL17 [32], which is also associated with tissue inflammation. NOD-like receptors (NLRs) are intracellular pattern recognition receptors that function by forming signaling complexes, including NOD signalosomes and inflammasomes [33]. The signaling pathway involving AGE and the receptor for AGE promotes the production of reactive oxygen species through NADPH oxidase. This in turn leads to endoplasmic reticulum stress, inflammation, and kidney fibrosis [34]. This study substantiates the findings of previous studies, underscoring the involvement of inflammatory responses and regulation in DN progression. To evaluate the impact of IRDEG expression on DN development, we performed GSEA to examine the expression patterns of all genes and their associated biological processes in DN and normal samples obtained from Dataset-DN. GSEA highlighted the enrichment of pathways, such as MET-mediated cell motility, collagen fibril assembly, and diseases related to glycosaminoglycan metabolism in the DN group, characterized by high IRDEG expression. In contrast, the normal group, with low IRDEG expression, showed enrichment in metabolism-related pathways, including propanoate and amino acid metabolism, as well as extracellular matrix (ECM) organization and structural components. DN, marked by elevated IRDEG expression, is linked with increased ECM deposition and renal fibrosis [35]. Notably, collagen, a well-studied ECM protein, serves as a biomarker for fibrogenesis in renal diseases, including DN [36]. The development of DN is closely associated with metabolic disorders [37].

To identify potential IRGs associated with DN, we employed two distinct algorithms (LASSO and SVM) along with logistic regression analysis on the 17 IRDEGs. Consequently, we identified five potential candidate genes: *CASP3, IFNAR2, LGALS9, NR4A1*, and *SST*. In DN samples, NR4A1 and SST exhibited downregulated expression, while *CASP3, IFNAR2*, and *LGALS9* displayed upregulated expression when compared with that in healthy samples. Furthermore, ROC curves confirmed that CASP3 and IFNAR2 exhibited relatively high accuracy, had a moderate degree of accuracy, and SST diagnostic accuracy was comparatively lower. However, upon performing IHC and RT-PCR analyses, we observed significant differential expression only in CASP3, LGALS9, and SST between healthy and DN samples, while IFNAR2 or NR4A1 did not exhibit significant differences. This suggests that CASP3, LGALS9, and SST may be reliable IRGs for DN.

CASP3, encoding caspase 3, is a cysteine–aspartic acid protease essential for the execution phase of cell death. Caspase-3 and Bax/Bcl-2 are important apoptosis markers. Recent research has unveiled the bidirectional interaction of apoptosis and pyroptosis with the innate immune system [38]. A study by Liu et al. [39] showed a significant increase in caspase-3 expression in the renal tissues of mice with STZ-induced diabetes compared to the control group. In line with our results, differential analysis demonstrated high CASP3 expression in the DN group; however, IHC analysis of human renal tissue and RT-PCR in mouse renal tissue revealed downregulation in CASP3. Shahzad et al. [40] demonstrated that caspase-3 loss did not protect mice against DN, implying that caspase-3-dependent cell death has a minor impact. The *LGALS9* gene encodes galectin-9, a member of the animal lectin family with conserved carbohydrate recognition domains showing strong affinity for β-galactosides. Galectin-9 plays a regulatory role in processes, such as proliferation, differentiation, apoptosis, and immunomodulation in various tissues [41]. Previous studies have documented the expression of galectin-9 in various immune cells [42]. Recent studies have highlighted elevated levels of Gal-9 in autoimmune diseases, tumors, chronic renal disease, type 2 diabetes, and other conditions [43,44]. Notably, individuals with type 2 diabetes and chronic renal failure exhibit elevated serum concentrations of galectin-9 [42]. In this study, LGALS9 was also overexpressed in the DN group, aligning with earlier research. SST, a widely recognized neuropeptide encoded by the *SST* gene, is distributed extensively across the brain [45]. Research suggests that the growth hormone-insulin-like growth factor–SST system may play a crucial role in nephropathies, including DN [46]. In experimental DN, octreotide, a nonselective long-term SST counterpart, is likewise helpful in suppressed circulating GH levels [46]. Analogs of SST have positive effects on DN similar to those of ACEI [47]. These results are consistent with those of previous studies. Currently, research on *CASP3, LGALS9,* and *SST* in the context of DN is limited. Therefore, further exploration is needed to reveal the molecular mechanisms and physiological functions of these key genes.

Immune infiltration analysis unveiled significant differences in the abundance of 21 distinct immune cell types between the high- and low-risk groups within the diagnostic model of the DN dataset. Furthermore, an association analysis between the infiltration abundance of these 21 immune cell types and the expression of the five key genes (*CASP3, IFNAR2, LGALS9, NR4A1,* and *SST*) revealed a significant correlation between gene expression and immune cell content. Specifically, most immune cells positively correlated with the expression of *CASP3, IFNAR2*, and *LGALS9*. A growing body of research suggests that immunological and inflammatory mechanisms are involved in the initiation and development of DN. Immune cells involved in both innate and adaptive immunity, such as mast cells, neutrophils, T and B lymphocytes, macrophages, and dendritic cells, all play a critical role in the pathological progression of DN [5,6,30,48]. One study provided evidence that the fundamental immunopathological mechanisms underlying diabetic kidney damage involve the abnormal infiltration and activation of T cells (CD3^+^, CD4^+^, and CD8^+^) within the renal interstitium [49]. Upon activation, B cells produce antibodies, inflammatory cytokines, immune complexes, and complement factors. These components have the potential to infiltrate the glomeruli and induce damage to renal tissues [50]. B lymphocytes also assist in the progression of DN. Elevated mast cell numbers and degranulation have been observed in renal biopsies of individuals with type 2 diabetes at different stages of nephropathy, which may induce renal inflammation and fibrosis, thus contributing to DN [51]. Using single-nucleus RNA sequencing, a study identified 347 leukocytes in the early stages of human DN, with T cells accounting for 49 %, B cells for 21 %, monocytes for 23 %, and plasma cells for 7 % of the total population [52]. Consistent with our findings, various T cell subsets, B cells, mast cells, and other immune cells have been implicated in the pathogenesis of DN. Macrophages constitute a significant proportion of the innate immune cells within the renal tissues of patients with DN. These macrophages accumulate in both the glomeruli and interstitium, and the extent of interstitial infiltration has been shown to correlate with the progression of renal function decline [53]. Recent research has focused on the role of M1/M2 macrophage polarization in the development of DN [54]. M1 macrophages produce proinflammatory mediators, such as TNF-a, MCP-1, iNOS, proteases, and other proinflammatory cytokines, aggravating inflammation, and contributing to the pathophysiology of DN [55]. M2 macrophages contribute to wound healing and anti-inflammatory responses by secreting cytokines, such as IL-10 and IL-4 [56]. Based on our hypothesis, individuals in the high group were more likely to develop substantial DN than those in the low group. Finally, we verified the expression of the five key genes through IHC in human kidney tissue and RT-PCR in mouse kidney tissue, and found that the expression of *CASP3, LGALS9,* and *SST* differed significantly between the control and DN groups.

However, the present study has some limitations. First, the sample size of our clinical cohort may have been inadequate for validating the expression patterns of all five key genes. Secondly, different pathogenic phases of DN may have influenced the findings of this study. Finally, the markers associated with the IRGs in patients with DN identified in this study were based on data from public databases. In summary, the identified IRGs and their associated pathways provided valuable insights into the onset and progression of DN. Nevertheless, further investigation is required to elucidate the biological activities and molecular mechanisms of these genes.

## 5 Conclusion

In conclusion, three genes (*CASP3, IFNAR2,* and *LGALS9*) were identified as potential IGRs in patients with DN. Additionally, this research has shed light on novel molecular pathways in patients with DN, which may pave the way for new treatment methods based on anti-inflammatory effects in the clinical management of DN in the near future.

## CRediT authorship contribution statement

**Jinxiu Deng:** Writing – original draft, Validation, Formal analysis. **Peiwen Wu:** Writing – review & editing, Supervision, Funding acquisition.

## Data availability

The datasets presented in this study can be found in online repositories. The names of the repository/repositories and accession number(s) can be found in the article.

## Funding

This study was supported by the Startup Fund for scientific research, 10.13039/501100013795Fujian Medical University (Grant number: 2019QH1208) and Joint Funds for the Innovation of Science and Technology, Fujian Province (No.2021Y9106).

## Declaration of competing interest

The authors declare that they have no known competing financial interests or personal relationships that could have appeared to influence the work reported in this paper.
