# Supplementary material for: Integrated bioinformatics analysis and in vivo validation of potential immune-related genes linked to diabetic nephropathy
Source: Heliyon. 2024 Nov 5;10(21):e40151. doi: 10.1016/j.heliyon.2024.e40151 (PMC11582746; doi:10.1016/j.heliyon.2024.e40151)
Supplement: Multimedia component 1 [file mmc1.docx]

### Table S1. mRNA–miRNA interaction network nodes.

| mRNA | miRNA | mRNA | miRNA |
| --- | --- | --- | --- |
| *CASP3* | hsa-miR-4795-3p | *IFNAR2* | hsa-miR-373-5p |
| *CASP3* | hsa-miR-133a-5p | *IFNAR2* | hsa-miR-616-5p |
| *CASP3* | hsa-miR-12125 | *IFNAR2* | hsa-miR-519a-3p |
| *CASP3* | hsa-miR-4500 | *IFNAR2* | hsa-miR-30d-5p |
| *CASP3* | hsa-miR-421 | *IFNAR2* | hsa-miR-30b-5p |
| *CASP3* | hsa-miR-4694-3p | *IFNAR2* | hsa-miR-30a-5p |
| *CASP3* | hsa-let-7c-5p | *IFNAR2* | hsa-miR-30c-5p |
| *CASP3* | hsa-let-7i-5p | *IFNAR2* | hsa-miR-30e-5p |
| *CASP3* | hsa-let-7a-5p | *IFNAR2* | hsa-miR-6765-3p |
| *CASP3* | hsa-let-7f-5p | *IFNAR2* | hsa-miR-4722-5p |
| *CASP3* | hsa-miR-98-5p | *IFNAR2* | hsa-miR-6732-3p |
| *CASP3* | hsa-let-7e-5p | *NR4A1* | hsa-miR-656-3p |
| *CASP3* | hsa-let-7g-5p | *NR4A1* | hsa-miR-4719 |
| *CASP3* | hsa-let-7b-5p | *NR4A1* | hsa-miR-608 |
| *CASP3* | hsa-miR-182-3p | *NR4A1* | hsa-miR-4651 |
| *CASP3* | hsa-miR-6795-5p | *NR4A1* | hsa-miR-4801 |
| *CASP3* | hsa-miR-6509-3p | *NR4A1* | hsa-miR-3190-3p |
| *CASP3* | hsa-miR-6887-5p | *NR4A1* | hsa-miR-1277-5p |
| *CASP3* | hsa-miR-4768-5p | *NR4A1* | hsa-miR-200b-5p |
| *CASP3* | hsa-let-7d-5p | *NR4A1* | hsa-miR-200a-5p |
| *CASP3* | hsa-miR-187-5p | *NR4A1* | hsa-miR-3152-3p |
| *CASP3* | hsa-miR-4458 | *NR4A1* | hsa-miR-4731-3p |
| *CASP3* | hsa-miR-6833-3p | *NR4A1* | hsa-miR-124-3p |
| *CASP3* | hsa-miR-3529-3p | *NR4A1* | hsa-miR-506-3p |
| *CASP3* | hsa-let-7c-3p | *NR4A1* | hsa-miR-4666a-5p |
| *CASP3* | hsa-miR-1184 | *NR4A1* | hsa-miR-215-3p |
| *CASP3* | hsa-miR-4698 | *NR4A1* | hsa-miR-12129 |
| *CASP3* | hsa-miR-382-5p | *NR4A1* | hsa-miR-4533 |
| *CASP3* | hsa-miR-6855-5p | *NR4A1* | hsa-miR-4778-3p |
| *CASP3* | hsa-miR-3170 | *SST* | hsa-miR-9500 |
| *CASP3* | hsa-miR-6512-5p | *SST* | hsa-miR-335-3p |
| *CASP3* | hsa-miR-601 | *SST* | hsa-miR-582-5p |
| *IFNAR2* | hsa-miR-1236-3p | *SST* | hsa-miR-4328 |
| *IFNAR2* | hsa-miR-4659a-3p | *SST* | hsa-miR-590-3p |
| *IFNAR2* | hsa-miR-4659b-3p | *SST* | hsa-miR-4257 |
| *IFNAR2* | hsa-miR-6515-3p | *SST* | hsa-miR-4775 |
| *IFNAR2* | hsa-miR-10524-5p | *SST* | hsa-miR-6822-5p |
| *IFNAR2* | hsa-miR-371b-5p | *SST* | hsa-miR-197-3p |
| *IFNAR2* | hsa-miR-519c-3p | *SST* | hsa-miR-6894-5p |
| *IFNAR2* | hsa-miR-519b-3p |  |  |

### TF, transcription factor

### Table S2. mRNA–TF interaction network nodes.

| mRNA | TF | mRNA | TF |
| --- | --- | --- | --- |
| *CASP3* | BRD2 | *IFNAR2* | SPI1 |
| *CASP3* | BRD4 | *IFNAR2* | SRF |
| *CASP3* | CEBPA | *IFNAR2* | STAT1 |
| *CASP3* | CTCF | *IFNAR2* | TCF12 |
| *CASP3* | E2F1 | *IFNAR2* | TCF3 |
| *CASP3* | EP300 | *IFNAR2* | TFAP4 |
| *CASP3* | ERG | *IFNAR2* | ZNF263 |
| *CASP3* | ETS1 | *LGALS9* | CEBPB |
| *CASP3* | FLI1 | *LGALS9* | MYB |
| *CASP3* | FOXA1 | *NR4A1* | BRD2 |
| *CASP3* | FOXA2 | *NR4A1* | CTCF |
| *CASP3* | GABPA | *NR4A1* | CTCFL |
| *CASP3* | GATA1 | *NR4A1* | E2F6 |
| *CASP3* | HDAC1 | *NR4A1* | EGR1 |
| *CASP3* | KLF1 | *NR4A1* | ELF1 |
| *CASP3* | KLF5 | *NR4A1* | EP300 |
| *CASP3* | MAZ | *NR4A1* | ERG |
| *CASP3* | RUNX3 | *NR4A1* | ETS1 |
| *CASP3* | ZNF263 | *NR4A1* | FLI1 |
| *IFNAR2* | BRD2 | *NR4A1* | FOXP2 |
| *IFNAR2* | BRD4 | *NR4A1* | GLIS1 |
| *IFNAR2* | CDK9 | *NR4A1* | IRF1 |
| *IFNAR2* | CEBPB | *NR4A1* | KLF1 |
| *IFNAR2* | CTCF | *NR4A1* | KLF4 |
| *IFNAR2* | E2F1 | *NR4A1* | KLF5 |
| *IFNAR2* | E2F6 | *NR4A1* | KLF9 |
| *IFNAR2* | EBF1 | *NR4A1* | LMO2 |
| *IFNAR2* | ELF1 | *NR4A1* | MAZ |
| *IFNAR2* | EP300 | *NR4A1* | MECOM |
| *IFNAR2* | FLI1 | *NR4A1* | MYB |
| *IFNAR2* | FOXA1 | *NR4A1* | MYOD1 |
| *IFNAR2* | FOXA2 | *NR4A1* | POLR2A |
| *IFNAR2* | GABPA | *NR4A1* | RUNX1 |
| *IFNAR2* | GATA1 | *NR4A1* | RUNX1T1 |
| *IFNAR2* | GRHL2 | *NR4A1* | SP1 |
| *IFNAR2* | HDAC2 | *NR4A1* | SPI1 |
| *IFNAR2* | MAX | *NR4A1* | STAG1 |
| *IFNAR2* | MAZ | *NR4A1* | TCF12 |
| *IFNAR2* | MYOD1 | *NR4A1* | ZBTB7A |
| *IFNAR2* | NR2F2 | *NR4A1* | ZNF384 |

### TF, transcription factor

### Table S3. mRNA–Drug interaction network nodes.

| mRNA | Drug | mRNA | Drug |
| --- | --- | --- | --- |
| *CASP3* | CELECOXIB | *CASP3* | TELEOCIDIN B |
| *CASP3* | SPERMINE | *IFNAR2* | SIFALIMUMAB |
| *CASP3* | PACLITAXEL | *IFNAR2* | PEGINTERFERON ALFA-2A |
| *CASP3* | CHEMBL483849 | *IFNAR2* | INTERFERON ALFA-2B |
| *CASP3* | WITHAFERIN A | *IFNAR2* | INTERFERON ALFACON-1 |
| *CASP3* | IPRIFLAVONE | *IFNAR2* | PEGINTERFERON BETA-1A |
| *CASP3* | 1,4-DICHLOROBENZENE | *IFNAR2* | INTERFERON BETA-1B |
| *CASP3* | EMRICASAN | *IFNAR2* | ANIFROLUMAB |
| *CASP3* | CHEMBL376616 | *IFNAR2* | ALBINTERFERON ALFA-2B |
| *CASP3* | CHEMBL1210769 | *IFNAR2* | PEGINTERFERON ALFA-2B |
| *CASP3* | NAPHTHOQUINONE | *IFNAR2* | INTERFERON BETA-1A |
| *CASP3* | CHEMBL560532 | *IFNAR2* | INTERFERON ALFA-N3 |
| *CASP3* | CHEMBL312032 | *IFNAR2* | INTERFERON ALFA-2A |
| *CASP3* | KOBOPHENOL A | *LGALS9* | LACTOSE, ANHYDROUS |
| *CASP3* | CHEMBL1493528 | *NR4A1* | LEVODOPA |
| *CASP3* | CHEMBL546865 | *NR4A1* | NICOTINE |
| *CASP3* | STAUROSPORINE | *NR4A1* | CHEMBL35482 |
| *CASP3* | CHEMBL1504679 | *NR4A1* | HALOPERIDOL |
| *CASP3* | CHEMBL584668 | *NR4A1* | IONOMYCIN |
| *CASP3* | CHEMBL299853 | *NR4A1* | MORPHINE |
| *CASP3* | XANTHOANGELOL B | *NR4A1* | CYTOSPORONE B |
| *CASP3* | CHEMBL600313 | *NR4A1* | ACETYLCYSTEINE |
| *CASP3* | CHEMBL412603 | *NR4A1* | CHEMBL547833 |
| *CASP3* | CHEMBL375563 | *NR4A1* | ETOPOSIDE PHOSPHATE |
| *CASP3* | CHEMBL546170 | *SST* | GANCICLOVIR |
| *CASP3* | DIACEREIN | *SST* | STREPTOZOCIN |
| *CASP3* | CHEMBL576208 | *SST* | CAPTOPRIL |
| *CASP3* | CHEMBL378903 | *SST* | VALINOMYCIN |
| *CASP3* | PHATHALIMIDE | *SST* | LITHIUM |
| *CASP3* | CHEMBL484663 | *SST* | CYSTEAMINE |
| *CASP3* | CHEMBL428789 | *SST* | AMPHETAMINE |
| *CASP3* | PAC-1 |  |  |
